# Supplementary material for: β-Cardiac myosin hypertrophic cardiomyopathy mutations release sequestered heads and increase enzymatic activity
Source: Nat Commun. 2019 Jun 18;10:2685. doi: 10.1038/s41467-019-10555-9 (PMC6582153; doi:10.1038/s41467-019-10555-9)
Supplement: Supplementary file 1 — Supplementary Information [file 41467_2019_10555_MOESM1_ESM.pdf]

Supplementary Information to:

β-Cardiac Myosin Hypertrophic Cardiomyopathy Mutations Release Sequestered Heads and Increase Enzymatic Activity

Arjun S. Adhikari[1,2], Darshan V. Trivedi[1,2], Saswata S. Sarkar[1,2], Dan Song[1,2], Kristina B. Kooiker[1,2,3], Daniel Bernstein[2,3], James A. Spudich[1,2], Kathleen M. Ruppel[1,2,3]

1. Department of Biochemistry, Stanford University School of Medicine, Stanford, CA 94305, USA
2. Stanford Cardiovascular Institute, Stanford, CA 94305, USA
3. Department of Pediatrics (Cardiology), Stanford University School of Medicine, Stanford, CA 94305, USA

I. Supplementary Figures
II. Supplementary Tables
III. Supplementary Methods
IV. Supplementary Movies
V. Supplementary References

Supplementary Figures

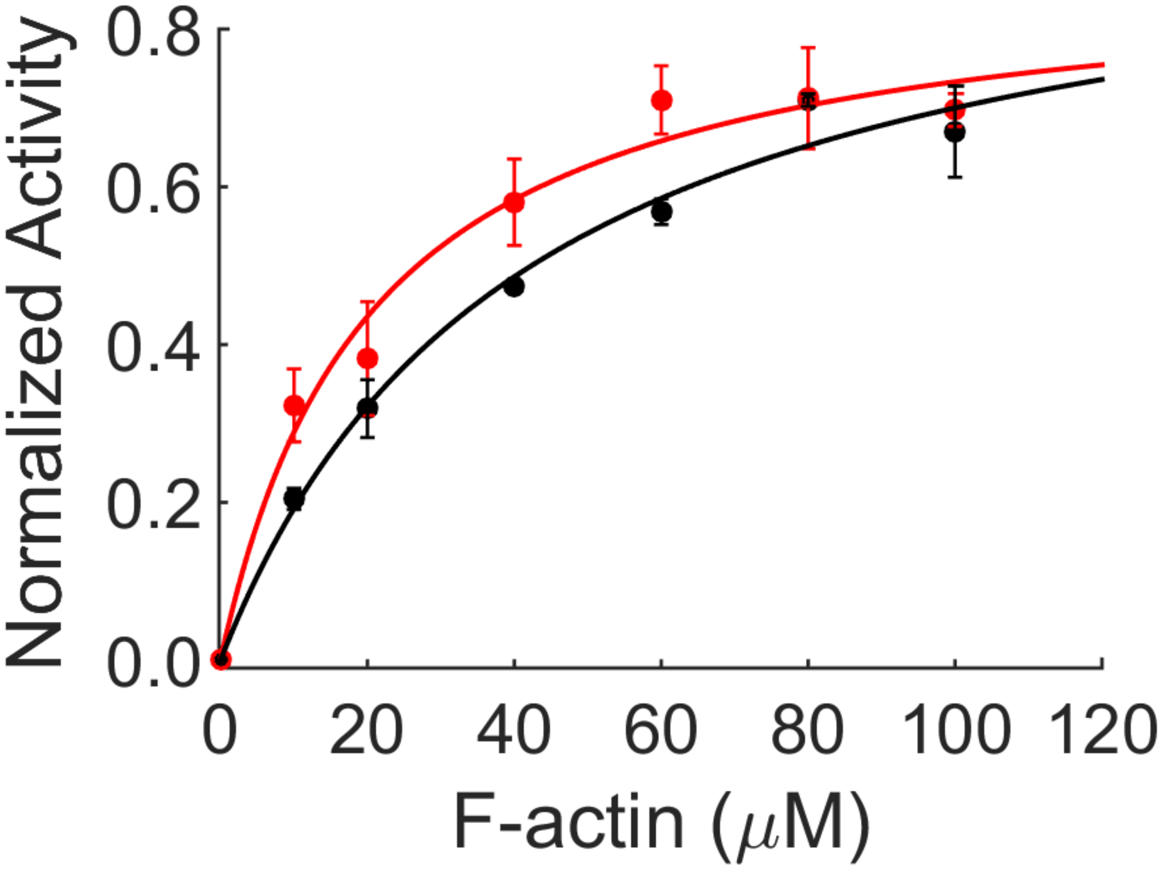

Supplementary Figure 1. **WT sS1 and 2-hep HMM have similar ATPase Activity.**
Actin-activated ATPase of WT sS1 (black) and 2-hep HMM (red). Error bars represent SEM for 3 biological replicates with 3 technical replicates.
K_{cat}sS1: K_{cat}2-hep = 1.1 ± 0.2 (P = 0.6)
K_{M}sS1: K_{M}2-hep = 2.0± 0.8 (P = 0.2)

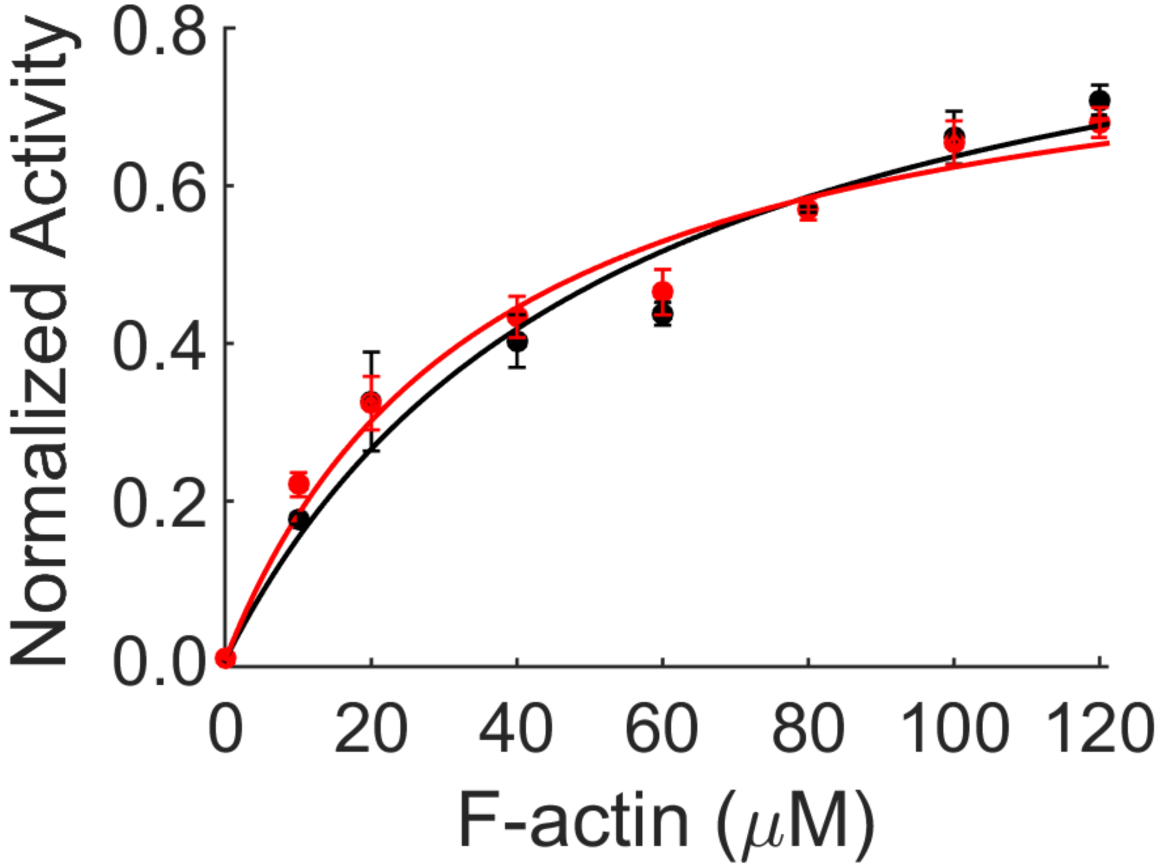

Supplementary Figure 2. R719W sS1 and 2-hep HMM have similar ATPase activity. Actin-activated ATPase of R719W sS1 (black) and 2-hep HMM (red). Error bars represent SEM for 2 biological replicates with 3 technical replicates.
K_{cat}sS1: K_{cat}2-hep = 1.2 ± 0.2 (P = 0.4)
K_MsS1: K_M2-hep = 1.5± 0.7 (P = 0.4)

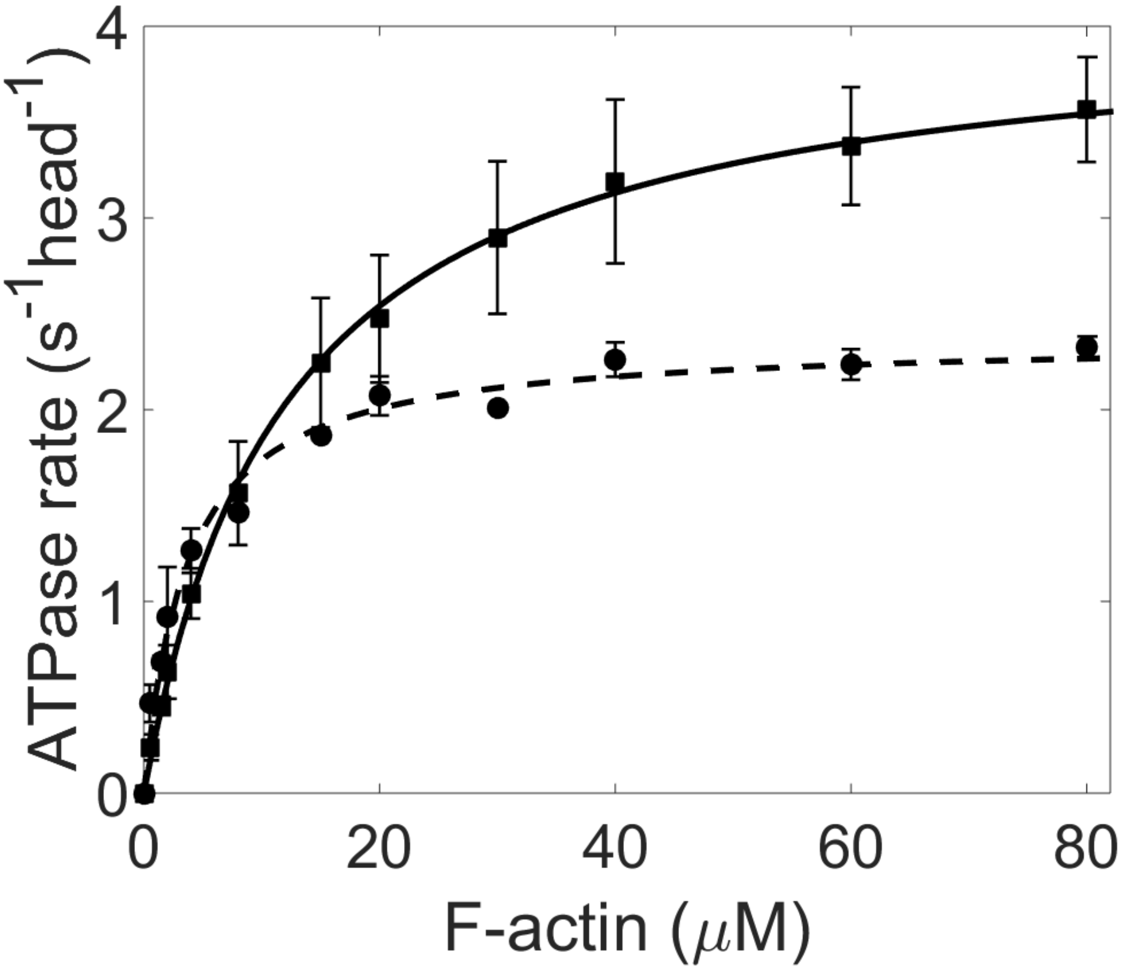

Supplementary Figure 3. **Effect of I457T mutation on HMM actin-activated ATPase kinetics.** Actin-activated ATPase of WT 2-hep HMM (circles and dashed line) vs I457T 2-hep HMM (squares and solid line) clearly shows that I457T has a higher k_{cat} compared to WT 2-hep HMM. Error bars represent SEM for 2 biological replicates with 3 technical replicates.

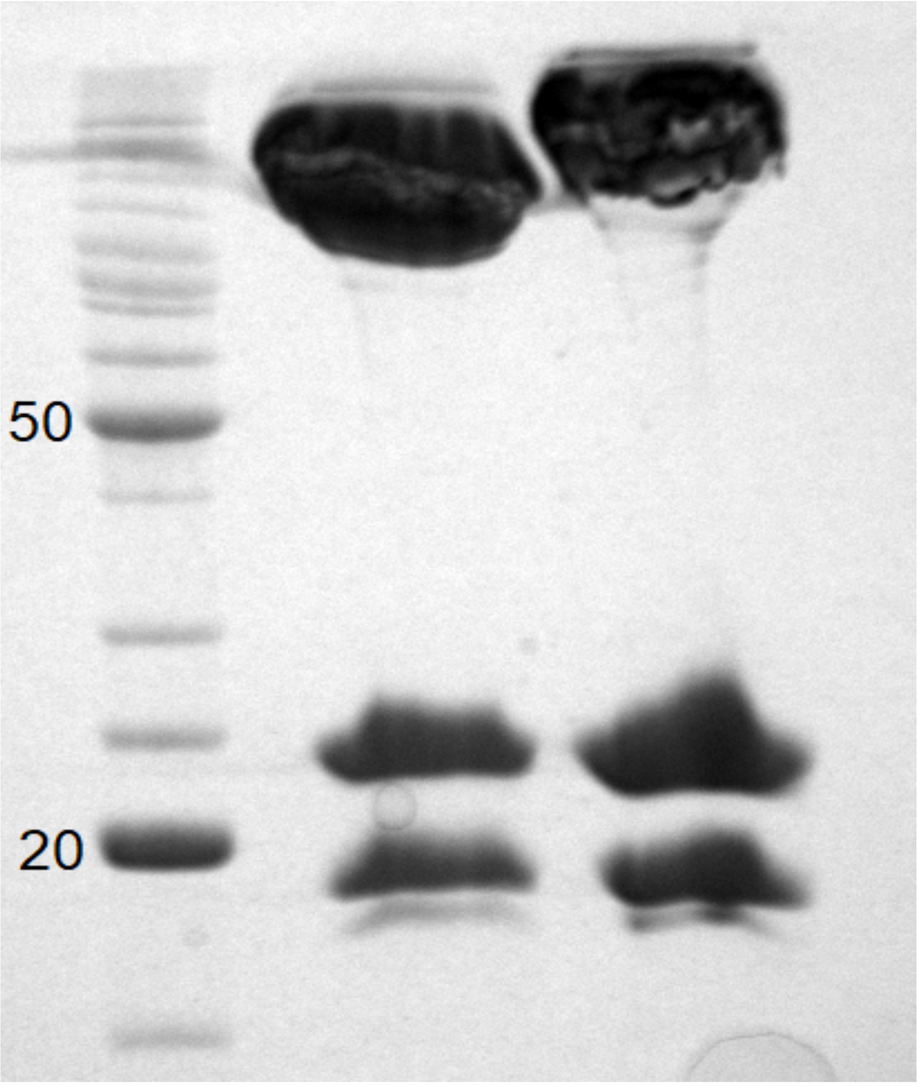

Supplementary Figure 4. SDS-PAGE of purified recombinant WT human β-cardiac 2-hep (middle lane) and 25-hep (right lane) HMM. BenchMark protein ladder standards (Invitrogen) are shown in the left lane. Note this is the same gel image as in Figure 3 in the main text but includes the protein molecular weight standards.

Supplementary Tables

	2-hep HMM k_{cat}	2-hep HMM k_M	25-hep HMM k_{cat}	25-hep HMM k_M	25-hep HMM:2-hep HMM k_{cat} ratio
**WT**	2.39 ± 0.06	3.6 ± 0.7	1.38 ± 0.06	9.3 ± 1.5	0.57 ± 0.03*
**R249Q**	1.69 ± 0.08	4.1 ± 0.6	1.63 ± 0.06	3.0 ± 0.3	0.96 ± 0.06^
**H251N**	3.31 ± 0.05	4.1 ± 0.2	2.95 ± 0.07	12.8 ± 0.9	0.91 ± 0.03*
**D382Y**	3.21 ± 0.06	2.2 ± 0.2	2.72 ± 0.12	7.1 ± 0.7	0.84 ± 0.04*
**R719W**	2.68 ± 0.04	5.0 ± 0.3	2.79 ± 0.07	9.7 ± 0.8	1.03 ± 0.03^
**D/R**	1.35 ± 0.02	1.5 ± 0.1	1.41 ± 0.03	3.3 ± 2	1.04 ± 0.04^
**I457T**	4.19 ± 0.33	12.4 ± 3	2.35 ± 0.31	33 ± 10	0.56 ± 0.09*

Supplementary Table 1. Actin-activated ATPase activity values for 2-hep HMM and 25-hep HMM. Raw data underlying the normalized values presented in Figures 3b, 4b, 4d, 5b, 5d, 6b, and 7d in the main text. Mean ± SEM of 2-3 biological replicates with 3 technical replicates for each protein prep. Note: D/R is the D382R/R719D double mutant. (*) denotes P-value <0.01; (^) denotes P-value >0.1 as compared to a k_{cat} ratio of 1 (no difference between 2-hep HMM and 25-hep HMM).

From the k_{cat} values in Supplementary Table 1, it is clear that H251N 2-hep HMM has a ~40% higher k_{cat} than WT 2-hep HMM, and R719W 2-hep HMM has a ~10% higher k_{cat} than WT 2-hep HMM. These values agree with previously published experiments on sS1 myosin with the same mutation (see Table S3).

In general, the K_M values varied somewhat between the different mutations, although no clear pattern emerged. This finding is similar to that reported by Ujfalusi et al. for a group of several different HCM- and DCM-causing β-cardiac myosin mutations[1]. In most cases, the K_M measured for the 2-hep HMM is lower than that of the corresponding 25-hep HMM. The reason for this is not entirely clear, and we are beginning to explore whether the presence of the leucine zipper after only 2 heptad repeats of the proximal S2 may affect the interaction of the two heads with the actin filament.

	2-hep	25-hep
**WT***	19 ± 3%	59 ± 7%
**R249Q^**	22 ± 1%	21 ± 3%
**H251N***	19 ± 1%	35 ± 4%
**D382Y****	18 ± 4%	32 ± 4%
**R719W^**	18 ± 3%	19 ± 7%
**D/R^**	22 ± 9%	27 ± 5%
**I457T***	18 ± 1%	44 ± 4%

Supplementary Table 2. **Slow phase fraction (SRX) of 2-hep and 25-hep HMM as measured using a single turnover mant ATPase assay.** Representative data is plotted in Figures 3a, 4a, 4c, 5a, 5c, 6a, and 7c in the main text. Table contains mean ± SEM of 2-3 biological replicates with 3 technical replicates for each protein prep. For the comparisons between the slow phase fraction of 2-hep and 25-hep, (*) denotes P-value <0.01; (**) denotes a P-value of 0.05; (^) denotes P-value >0.1

	2-hep slow fraction	2-hep slow rate	2-hep fast fraction	2-hep fast rate
**WT**	0.19 ± 0.03	0.002 ± 0.001	0.81 ± 0.03	0.02 ± 0.002
**R249Q**	0.22 ± 0.01	0.004 ± 0.001	0.78 ± 0.01	0.025 ± 0.003
**H251N**	0.19 ± 0.01	0.005 ± 0.002	0.81 ± 0.01	0.04 ± 0.02
**D382Y**	0.18 ± 0.04	0.003 ± 0.001	0.82 ± 0.04	0.02 ± 0.001
**R719W**	0.18 ± 0.03	0.002 ± 0.002	0.82 ± 0.03	0.025 ± 0.002
**D/R**	0.22 ± 0.09	0.002 ± 0.001	0.78 ± 0.09	0.02 ± 0.003
**I457T**	0.18 ± 0.01	0.002 ± 0.0004	0.82 ± 0.01	0.02 ± 0.01

Supplementary Table 3. Single turnover parameters for 2-hep HMM Representative data is plotted in Figures 3a, 4a, 4c, 5a, 5c, 6a, and 7c in the main text. Table contains mean ± SEM of 2-3 biological replicates with 3 technical replicates for each protein prep.

	25-hep slow fraction	25-hep slow rate	25-hep fast fraction	25-hep fast rate
**WT**	0.59 ± 0.07	0.003 ± 0.001	0.41 ± 0.07	0.02 ± 0.003
**R249Q**	0.21 ± 0.03	0.003 ± 0.002	0.79 ± 0.03	0.03 ± 0.006
**H251N**	0.35 ± 0.04	0.003 ± 0.001	0.65 ± 0.04	0.04 ± 0.02
**D382Y**	0.32 ± 0.04	0.003 ± 0.001	0.68 ± 0.04	0.02 ± 0.003
**R719W**	0.19 ± 0.07	0.003 ± 0.002	0.81 ± 0.07	0.02 ± 0.003
**D/R**	0.27 ± 0.05	0.006 ± 0.002	0.73 ± 0.05	0.03 ± 0.01
**I457T**	0.44 ± 0.04	0.003 ± 0.001	0.56 ± 0.04	0.03 ± 0.006

Supplementary Table 4. Single turnover parameters for 25-hep HMM
Representative data is plotted in Figures 3a, 4a, 4c, 5a, 5c, 6a, and 7c in the main text. Table contains mean ± SEM of 2-3 biological replicates with 3 technical replicates for each protein prep.

HCM mutation	Intrinsic force (F_{int})	Velocity (vel)	ATPase (k_{cat})
R403Q[2]	0.8 ± 0.1	1.2 ± 0.1	1.2 ± 0.1
R453C[3]	1.5 ± 0.1	0.8 ± 0.1	0.7 ± 0.1
R719W[4]	0.8 ± 0.1	1.2 ± 0.1	1.0 ± 0.1
R723G[4]	0.8 ± 0.1	1.1 ± 0.1	1.0 ± 0.1
G741R[4]	1.0 ± 0.1	1.0 ± 0.1	1.0 ± 0.1
H251N[5]	1.3 ± 0.1	1.4 ± 0.03	1.3 ± 0.1
D239N[5]	1.2 ± 0.1	1.8 ± 0.04	1.5 ± 0.1
D382Y	-	0.9 ± 0.02	1.2 ± 0.1
R249Q	-	0.5 ± 0.05	0.63 ± 0.1

Supplementary Table 5. Summary of mechano-chemical properties of myosin HCM mutations using the sS1 backbone.

Shown are the ratio of mutant:WT for the listed mechano-chemical parameters of sS1 myosin containing various HCM mutants. The sS1 myosin isolates the effect of the mutations on the biochemical/biomechanical function of the motor domain without having to account for the intramolecular head – head and head – tail interactions. Our previous work with R403Q, R453C, R719W, R723G, and G741R showed that, with the exception of the change in intrinsic force for R453C (50% increase), most of the gains in function for these mutations was less than 20%. We generally consider this a small change in the molecular assays. Moreover, for all of those mutations, at least one of the parameters showed a loss of function (except G741R, which showed no change for any of the measured parameters). Next, we measured early-onset mutations H251N and D239N and found that both these mutations led to increases in function for all the parameters (>20% and up to 90%) measured.

R249Q characterization

As seen in Supplementary Table 1, the actin-activated ATPase rate for R249Q 2-hep HMM is lower than that of the WT 2-hep HMM by 33 ± 4%. We see a similar decrease in ATPase using sS1 (Table S3). We further characterized the mutation using the in vitro motility assay using sS1 and found that the velocity was 50 ± 5% lower. However, we have also shown previously that R249Q weakens the binding between the S1 head and the S2 tail of myosin[7], and the ATPase data in this manuscript shows that R249Q's weakening of the S1-S2 interaction leads to increased activity of 25-hep HMM. These data suggest that even though the mutation may lead to a loss in the activity of individual myosin motors, it releases more myosin heads from the closed state making them available to interact with the actin in the sarcomere, thereby leading to hypercontractility.

D382Y characterization

The D382Y mutation results in k_{cat} values for 2-hep HMM that are 33 ± 4% higher compared to the WT 2-hep HMM. Our previous experiments using the in vitro motility assay showed that the velocity of D382Y was 10 ± 2% lower than the WT. In this case, we have a significant gain in function in the actin-activated ATPase rate, a very small decrease in the actin gliding velocity, and a significant decrease in the percentage of heads in the SRX. Overall, these data are consistent with the D382Y mutation causing hypercontractility at the molecular level.

Supplementary Methods

Protein Constructs, Expression, and Purification procedures:
Recombinant human β-cardiac myosin constructs: The human β-cardiac 2-hep HMM cDNA consists of a truncated version of MYH7 (residues 1-855), corresponding to S1 and the first two heptad repeats of S2, followed by a GCN4 leucine zipper[8] to ensure dimerization. This is further linked to a flexible GSG (Gly-Ser-Gly) linker, then an eGFP moiety followed by another GSG linker, and finally ending with an 8-residue (RGSIDTWV) PDZ-binding peptide. The human β-cardiac 25-hep HMM construct was similar to the 2-hep HMM, except that MYH7 was truncated after 25 heptad repeats (175 amino acids) of the S2 region (up to residue 1016). Mutations were introduced into a pBluescript KS-based plasmid containing the coding region for residues 1-808 of MYH7 using the Stratagene QuickChange site-directed mutagenesis protocol. Sequences of mutagenic primers are as follows:

R249Q sense: 5'-TGTTGCCCCAAAATGAATTTGAATGAATTTCCCGAAGCG-3'
R249Q antisense: 5'-CGCTTCGGGAAATTCATTCAAATTCATTTTGGGGCAACA-3'
H251N sense: 5'-TTCGGGAAATTCATTCGAATTAATTTTGGGGCAACAGGAAAG-3'
H251N anti-sense: 5'-CTTTCCTGTTGCCCCAAAATTAATTCGAATGAATTTCCCGAA-3'
D382R sense: 5'-CGGCACTGAAGAGGCTCGCAAGTCTGCCTACCTC-3'
D382R anti-sense: 5'-GAGGTAGGCAGACTTGCGAGCCTCTTCAGTGCCG-3'
D382Y sense: 5'-GACGGCACTGAAGAGGCTTATAAGTCTGCCTACCTCATG-3'
D382Y anti-sense: 5'-CATGAGGTAGGCAGACTTATAAGCCTCTTCAGTGCCGTC-3'
I457T sense: 5'-AGCAGCCACGCCAGTACTTCACGGGAGTCCTGGA-3'
I457T anti-sense: 5'-TCCAGGACTCCCGTGAAGTCTGGCGTGGCTGCT-3'
R719D sense: 5'-ATCCTCTACGGGGACTTCGATCAGAGGTATCGCATCCTG-3'
R719D anti-sense: 5'-CAGGATGCGATACCTCTGATCGAAGTCCCCGTAGAGGAT-3'
R719W sense: 5'-CTACGGGGACTTCTGGCAGAGGTATCGC-3'
R719W anti-sense: 5'-GCGATACCTCTGCCAGAAGTCCCCGTAG-3'

After sequencing to confirm the presence of the desired mutation and the absence of off-target mutations, the mutated MYH7 coding region was excised as an Age1-Mlu1 fragment and ligated into either pShuttleWT2hep or pShuttleWT25hep (intermediate shuttle vectors used in the pAdEasy Vector system, Qbiogene). Age1 cuts upstream of the start ATG and Mlu1 cuts uniquely near the 3' end of the coding sequence for the sS1 domain. Resultant plasmids were sequenced to confirm replacement of the WT sequence with the desired mutation prior to making the final adenoviral vectors.

Recombinant human β-cardiac 2-hep HMM and 25-hep HMM containing HCM-causing mutations were co-expressed with a FLAG-tagged human ventricular cardiac essential light chain (ELC) in C2C12 mouse myoblast cells using adenoviral vectors (pAd Easy system Qbiogene). C2C12 cells were grown at 37°C and 8% CO_2 in growth medium (DMEM + 10% fetal bovine serum + 1x pen-strep). 10 plates of confluent C2C12 cells were differentiated to myotubes by adding differentiation medium (DMEM + 2% horse serum + 1x pen-strep). The cells were differentiated for 2 days. Next, the cells were

infected with adenoviruses carrying the myosin sS1 and myosin FLAG-ELC in growth media ½ (DMEM + 5% fetal bovine serum + 1x pen-strep). The cells were infected for 4 days and then harvested. The media was removed from the cells, and they were washed with ice cold PBS. Then 1 ml of lysis buffer (20 mM imidazole pH7.5, 100 mM NaCl, 4 mM $MgCl_2$, 1 mM EGTA, 1 mM EDTA, 0.5% tween-20, 1 mM DTT, 3 mM ATP, 1 mM PMSF, 10% sucrose and Roche protease inhibitors) was added to each plate and the cells were harvested by scraping. The cells were lysed using a dounce homogenizer, and the lysate clarified by centrifugation at 23,000 rpm for 20 min using a Ti60 ultracentrifuge rotor. The supernatant was then incubated with 75 µL of anti-FLAG resin per 15 cm dish for 1.5 - 2 hours at 4°C. This allowed the HMM to bind to the anti-FLAG resin.

Next, the mouse RLC was stripped from the protein with 0.5% Triton X-100, 5 mM CDTA, 200 mM KCl and 20 mM Tris pH 7.5 at 4˚C for 75 min. Human RLC binding to the heavy chain was performed by incubation of an excess amount of recombinant human cardiac RLC for 2 hr at 4˚C. The protein was eluted from the anti-FLAG resin by incubating it with TEV protease overnight at 4˚C, and it was purified the next day using anion exchange chromatography. Analysis of a 15% SDS PAGE gel confirmed a 1:1:1 stoichiometric complex of HMM heavy chain with both human light chains. The RLC on the human HMM is unphosphorylated. All experiments presented in this manuscript were performed using unphosphorylated HMM.

RLC: Human ventricular regulatory light chain (RLC, MYL2) was purified using either a standard bacterial expression and affinity purification protocol, or via an inclusion body protocol for higher yields. Both protocols are below

Standard Protocol: RLC was expressed separately in E. coli using a pET-28b vector. The RLC construct had an N-terminal His-tag followed by TEV protease site. Bacterial cells containing the recombinant DNA were grown, induced and harvested as described by the manufacturer (Qiagen, Germany). The cells were then lysed using an Emulsiflex (EmulsiFlex-C5, Avestin, Canada) and the lysate was clarified by centrifugation at 35,000Å~g for 30 min. The supernatant was then loaded on a Ni-NTA column (GE) on a FPLC. A step and gradient protocol was followed to wash and elute the protein of interest. The column was extensively washed in steps with buffer containing 20 mM, 40 mM, 80 mM and 100 mM imidazole. Elution with a gradient of 100-400 mM imidazole was then performed followed by a final wash with 500 mM imidazole. All fractions were analyzed by SDS-PAGE and fractions containing the purest protein were pooled together.

Inclusion body protocol: Human ventricular regulatory light chain (RLC, MYL2) was expressed separately in Rosetta (DE3) pLysS E. coli bacteria (Novagen) using a pET-28b vector. The RLC construct had an N-terminal His6-tag followed by a tobacco etch virus (TEV) protease recognition site. Protein expression was induced with 0.8 mM IPTG for 3 h at 37°C when OD_{600} reached 0.6. Cell pellets were lysed by sonication in denaturing lysis buffer (20 mM Tris at pH 7.5, 1 M NaCl, 40 mM Imidazole, 1 mM PMSF, 6 M Urea, and 5 mM BME) to release the RLC from inclusion bodies. Sonicated

lysate was clarified by ultracentrifugation for 40 min at 35k rpm in a Ti 60 rotor (Beckman Coulter). The clarified supernatant was dialyzed overnight at 4°C against refolding buffer, which is essentially lysis buffer without urea. On the next day, dialyzed supernatant was further clarified by ultracentrifugation. The supernatant was bound to Ni-NTA resin (Qiagen) pre-equilibrated with the refolding buffer for 1 h and washed sequentially with 10x column volume of the refolding buffer and salt-reduction buffer (20 mM Tris at pH 7.5, 300 mM NaCl, 40 mM Imidazole, and 5 mM BME). Proteins were then eluted with elution buffer (20 mM Tris at pH 7.5, 300 mM NaCl, 250 mM Imidazole, and 5 mM BME) in fractions. Peak fractions were collected and dialyzed overnight at 4°C against storage buffer (20 mM Tris at pH 7.5, 300 mM NaCl, and 5 mM BME). The dialyzed protein was first spun down at 95k rpm for 5 min in a TLA-110 rotor (Beckman Coulter) to remove precipitate. The supernatant containing RLC purified to near homogeneity as confirmed by SDS-PAGE was flash frozen in aliquots with liquid nitrogen and stored at -80°C.

PDZ18: PDZ18 is a chimeric protein consisting of a human erbin PDZ domain and a fibronectin domain. It is engineered to bind to an eight-residue peptide (RGSIDTWV) with a single-nanomolar affinity (ePDZ-b1; Huang et al. 2009)[9]. The pHFT2 expression vector carrying PDZ18 with an N-terminal His10-FLAG tag and a tobacco etch virus (TEV) protease recognition site right after the tag (Huang et al. 2009) was used to express the proteins in Rosetta (DE3) pLysS *E. coli* bacteria (Novagen). Briefly, protein expression was induced with 0.8 mM IPTG for 3 h at 37°C when OD_{600} reached 0.6. Cell pellets were lysed by sonication in lysis buffer (50 mM Tris at pH 7.5, 500 mM NaCl, 40 mM Imidazole, 1 mM PMSF, and 5 mM BME), and supernatants were clarified by ultracentrifugation for 15 min at 95k rpm in a TLA-110 rotor (Beckman Coulter). The clarified supernatant was bound to Ni-NTA resin (Qiagen) for 1 h and washed sequentially with 10x column volume of lysis buffer and salt-reduction buffer (50 mM Tris at pH 7.5, 300 mM NaCl, 40 mM Imidazole, and 5 mM BME). Proteins were then eluted with elution buffer (50 mM Tris at pH 7.5, 300 mM NaCl, 250 mM Imidazole, and 5 mM BME) in 1 ml fractions. Peak fractions were collected and treated with His-tagged TEV protease while dialyzing overnight at 4°C against storage buffer (20 mM Tris at pH 7.5, 300 mM NaCl, 10% glycerol and 5 mM BME). The dialyzed protein was first spun down at 14k rpm for 30 min to remove minimal amount of precipitate. The supernatant was then incubated with Ni-NTA resin again for 1 h. The flow through containing untagged PDZ18 was collected, flash frozen in aliquots with liquid nitrogen and stored at -80°C.

<u>*Actin activated ATPase assay:*</u> Only freshly prepared HMM was used for ATPase assays. Since we aimed to elucidate whether HCM mutations alter the intramolecular interactions of myosin, we always prepared the 2-hep HMM and 25-hep HMM for each mutation in parallel, and the assays were performed on both proteins simultaneously. To prepare F-actin, bovine cardiac G-actin (gift of MyoKardia) was dialyzed extensively into ATPase buffer to remove any residual ATP. Actin concentration was then measured using absorbance at 290 nm in a spectrophotometer. The steady-state actin-activated ATPase activities of the WT 2-hep and 25-hep HMM and mutant human β-cardiac 2-hep and 25-hep HMMs were determined using a colorimetric assay to measure inorganic

phosphate production at various time points (0 - 30 min) from a solution containing myosin (0.01 mg ml^{-1}), ATP and varying concentrations of actin filaments (0 - 80 μM) [10]. All measurements were made at 23°C. The time-dependent rate for each actin concentration was calculated by fitting the phosphate signal as a function of time to a linear function. The slope was then converted to activity units normalized to a single myosin head. Kinetic parameters (i.e. k_{cat}) were extracted from the data by fitting the activity at each actin concentration to the Michaelis-Menten equation to determine maximal activity using the curve fitting toolbox in MatLab[11]. The errors in the fitted values were determined using 100 bootstrap iterations.

Microscale thermophoresis for sS1-S2 binding affinity: To assess if the mutations at the S1-S1 interface altered the interaction between S1 and S2, we used microscale thermophoresis (MST). To study this interaction, we used freshly prepared 2-hep HMM with its C-terminal eGFP, and unlabeled S2 expressed in and purified from bacteria (amino acids 839-968)[7]. The proximal S2 construct includes the first 126 amino acids of S2 and begins 4 residues before the end of S1. Due to the low affinity between S1 and S2, the S2 fragment was concentrated to >300 μM to get a binding curve. Both proteins were dialyzed into MST assay buffer (10 mM Imidazole pH7.5, 100 mM KCl, 1 mM EDTA, 2 mM $MgCl_2$, 1 mM DTT, 500 μM ADP and 0.05% tween). Before using them for the assay, both proteins were centrifuged at 100,000 rpm in a TLA 100 rotor for 20 min to remove any aggregates. For the assay, we used 16 serial dilutions of S2 myosin starting at > 300 μM, with a 3-fold dilution for each subsequent sample. The 2-hep HMM was kept constant at 50 nM. The samples were loaded into NT.115 premium treated capillaries and incubated at 23°C for 45 minutes in the dark. All MST data was recorded at 23°C. The 2-hep HMM-S2 interaction was followed by monitoring the eGFP fluorescence. A blue LED at 30% excitation power (BLUE filter; excitation 460-480 nm, emission 515-530 nm) and IR-Laser power at 60% was used. Data analysis was performed with the software NTAffinityAnalysis (Nanotemper Technologies) where the binding isotherms were derived from the raw fluorescence data. The binding isotherms were then fitted using Matlab, with the Hill equation for cooperativity, to estimate an apparent dissociation constant (K_D), using a linear regression method. There were preparation-to-preparation differences in the binding affinity, and the WT K_D ranged between 35-50 μM; however, the relative differences in binding affinities between the WT and mutant myosins were constant.

Single turnover experiments: Single turnover experiments were performed in a fluorescence plate reader (Tecan model – Infinite M200 PRO). Experiments were performed with the WT and mutant versions of 25-hep HMM and 2-hep HMM constructs of human ß-cardiac myosin as described[12]. Because of the slow rates of the myosin basal ATPase and SRX-based nucleotide release along with difficulties in expressing large quantities of human cardiac myosin, a low volume, plate-based measurement is an apt choice for measuring the single turnover rates. Appropriate controls and comparisons to a stopped-flow measurement were performed by Anderson et al.[12] to ensure that we are not missing any fast phase in the plate-based measurement. Briefly, these experiments were performed in a 96-well plate (Greiner polypropylene microplate) by mixing HMM in a buffer containing 10 mM Tris pH 7.5, 4 mM $MgCl_2$, 1 mM EDTA, 1 mM DTT and 5 mM KAc with 2'-(or-3')-*O*-(*N*-Methylanthraniloyl) adenosine 5'-

triphosphate (mant-ATP, Thermo Fischer Scientific) at a final concentration of 100 nM. After 10 s, 2 mM ATP was added, followed by measuring the fluorescence signal at 470 nm after excitation at 405 nm. Fluorescence was recorded every ~2 s for 16 min total and the traces were normalized and plotted[12]. The kinetic traces were fitted to a bi-exponential decay function which yielded the amplitudes and rates of the fast (DRX rate) and slow (SRX rate) phases.

Unloaded in vitro motility assay: Multi-channel flow chambers were constructed by mounting coverslips (VWR; No. 1.5) pre-coated with 0.1% nitrocellulose (Ladd Research Industries) and 0.1% collodion (Electron Microscopy Science) dissolved in amyl acetate (Sigma) on a glass slide (Gold Seal) using double-sided tape (Scotch). Reagents were sequentially flowed through the channels in the following order: (1) 10 µl of Assay Buffer (AB; 25 mM KCl, 4 mM $MgCl_2$, 25 mM Imidazole at pH 7.5, 1 mM EGTA, and 10 mM DTT); (2) 10 µl of 2 µM PDZ18 diluted in AB and incubated for 2 min; (3) 20 µl of 1 mg ml^{-1} BSA (Sigma) diluted in AB (ABBSA) to wash out unbound PDZ18 and block for non-specific binding; (4) 20 µl of the WT or mutant 2-hep HMM that contains a C-terminal affinity tag (RGSIDTWV), which can be recognized by PDZ18 on the surface for immobilization (Huang et al., 2009), and incubated for 5 min; (5) 10 µl of ABBSA to wash out unbound myosin; (6) 20 µl of a mixture of 1 – 5 nM bovine F-actin labeled with tetramethylrhodamine (TMR)-phalloidin (Invitrogen) diluted in ABBSA, 2 mM ATP (CalBiochem), and an oxygen scavenging system [0.4% glucose, 0.216 mg ml^{-1} glucose oxidase (CalBiochem), and 0.036 mg ml^{-1} catalase (CalBiochem)]. In general, one channel with wild-type and one channel with mutant cardiac myosin at a final concentration of 50 – 300 nM were prepared in parallel on the same slide. Time-lapse images were taken on a total internal reflection fluorescence microscope (Nikon Ti-E) coupled with a 100x objective (Nikon, NA 1.49) and an EMCCD camera (Andor) at 1 Hz with 300 ms exposure. At least three movies with a duration of 30 – 60 s were recorded for each channel. All experiments were conducted at 23°C. Filament tracking was performed using FAST (Fast Automated Spud Trekker) (Aksel et al. 2015), and velocities reported are the top 5% velocities (Aksel et al. 2015)[13].

Supplementary Movie 1: WT 2-hep HMM in-vitro motility

Supplementary Movie 2: I457T 2-hep HMM in-vitro motility

Supplementary References

1. Ujfalusi, Z. et al. Dilated cardiomyopathy myosin mutants have reduced force-generating capacity. *J Biol Chem* **293**, 9017-9029 (2018).
2. Nag, S. et al. Contractility parameters of human beta-cardiac myosin with the hypertrophic cardiomyopathy mutation R403Q show loss of motor function. *Sci Adv* **1**, e1500511 (2015).
3. Sommese, R.F. et al. Molecular consequences of the R453C hypertrophic cardiomyopathy mutation on human beta-cardiac myosin motor function. *Proc Natl Acad Sci U S A* **110**, 12607-12 (2013).
4. Kawana, M., Sarkar, S.S., Sutton, S., Ruppel, K.M. & Spudich, J.A. Biophysical properties of human beta-cardiac myosin with converter mutations that cause hypertrophic cardiomyopathy. *Sci Adv* **3**, e1601959 (2017).
5. Adhikari, A.S. et al. Early-Onset Hypertrophic Cardiomyopathy Mutations Significantly Increase the Velocity, Force, and Actin-Activated ATPase Activity of Human β-Cardiac Myosin. *Cell Reports* **17**, 2857-2864 (2016).
6. Alfares, A.A. et al. Results of clinical genetic testing of 2,912 probands with hypertrophic cardiomyopathy: expanded panels offer limited additional sensitivity. *Genetics in Medicine* **17**, 880-888 (2015).
7. Nag, S. et al. The myosin mesa and the basis of hypercontractility caused by hypertrophic cardiomyopathy mutations. *Nat Struct Mol Biol* **24**, 525-533 (2017).
8. Trybus, K.M., Freyzon, Y., Faust, L.Z. & Sweeney, H.L. Spare the rod, spoil the regulation: necessity for a myosin rod. *Proc Natl Acad Sci U S A* **94**, 48-52 (1997).
9. Huang, J., Nagy, S.S., Koide, A., Rock, R.S. & Koide, S. A peptide tag system for facile purification and single-molecule immobilization. *Biochemistry* **48**, 11834-6 (2009).
10. Trybus, K.M. Biochemical studies of myosin. *Methods.* **22**, 327-35 (2000).
11. De La Cruz, E.M. & Ostap, E.M. Kinetic and equilibrium analysis of the myosin ATPase. *Methods Enzymol* **455**, 157-92 (2009).
12. Anderson, R.L. et al. Deciphering the super relaxed state of human beta-cardiac myosin and the mode of action of mavacamten from myosin molecules to muscle fibers. *Proc Natl Acad Sci U S A* **115**, E8143-E8152 (2018).
13. Aksel, T., Yu, E.C., Sutton, S., Ruppel, K.M. & Spudich, J.A. Ensemble Force Changes that Result from Human Cardiac Myosin Mutations and a Small-Molecule Effector. *Cell Reports* **11**, 910-920 (2015).
