# Supplementary material for: β-Cardiac myosin hypertrophic cardiomyopathy mutations release sequestered heads and increase enzymatic activity
Source: Nat Commun. 2019 Jun 18;10:2685. doi: 10.1038/s41467-019-10555-9 (PMC6582153; doi:10.1038/s41467-019-10555-9)
Supplement: Supplementary file 5 — Description of Additional Supplementary Files [file 41467_2019_10555_MOESM5_ESM.docx]

**Title: Supplementary Movie 1**
**Description:** WT 2-hep HMM in-vitro motility (scale bar is 10 μm).

5 nM fluorescently labeled bovine cardiac F-actin moving over a surface of WT 2hep-HMM in the presence of 2 mM ATP. Time-lapse images were taken on a total internal reflection fluorescence microscope (Nikon Ti-E) coupled with a 100x objective (Nikon, NA 1.49) and an EMCCD camera (Andor) at 1 Hz with 300 ms exposure. At least three movies with a duration of 30 – 60 s were recorded for each channel. All experiments were conducted at 23°C.

**Title: Supplementary Movie 2**
**Description:** I457T 2-hep HMM in-vitro motility (scale bar is 10 μm).

5 nM fluorescently labeled bovine cardiac F-actin moving over a surface of I457T 2hep-HMM in the presence of 2 mM ATP. Time-lapse images were taken on a total internal reflection fluorescence microscope (Nikon Ti-E) coupled with a 100x objective (Nikon, NA 1.49) and an EMCCD camera (Andor) at 1 Hz with 300 ms exposure. At least three movies with a duration of 30 – 60 s were recorded for each channel. All experiments were conducted at 23°C.

EDITORIAL REQUESTS:
Your manuscript has been checked for clarity and against journal policies and formatting style. The issues listed below must be addressed. If using Microsoft Word, please use the tracked changes feature to make these changes.


***Please provide a point-by-point response to the list below with your submission.***
*Please see our responses below in italicized blue font.*

* Please supply the main figures as separate files.
*The main figures have been uploaded as individual tiff files. Note that only Fig 6 is different from the originally submitted figures.*

* Please provide us with a Source data File. This file should include all data underlying data of graphs, plots and full length gels/blots in a Source data File (See below for more information):

*The file “Adhikari et al Source Data File” has been uploaded. Please note that Fig. 1 contains only PyMol figures with the appropriate PDB file referenced; there is no data to supply separately.*


The source data file should, as a minimum, contain the raw data underlying all reported averages in graphs and charts, and uncropped versions of any gels or blots presented in the figures. To learn more about our motivation behind this policy, please see <https://www.nature.com/articles/s41467-018-06012-8.>

Within the source data file, each figure or table (in the main manuscript and in the Supplementary Information) containing relevant data should be represented by a single sheet in an Excel document, or a single .txt file or other file type in a zipped folder. Blot and gel images should be pasted in and labelled with the relevant panel and identifying information such as the antibody used. We also encourage you to include any other types of raw data that may be appropriate. An example source data file is available demonstrating the correct format:
<https://www.nature.com/documents/ncomms-example-source-data.xlsx>

The file should be labelled ‘Source Data’, with the title and a brief description included in your cover letter, and should be mentioned in all relevant figure legends using the template text below:
“Source data are provided as a Source Data file.”

*This information has been added to the relevant figure legends.*


TITLE PAGE


* Please edit the title so that it is 15 words or fewer and does not include punctuation.

*done*


MAIN TEXT

* The main text should include only the following sections: Introduction, Results, and optional Discussion, each of which must begin with a heading. All other section headings should be removed or renamed.

*? not an issue*


* Please use the present tense when discussing the current work in the Introduction.

*fixed*


* Please shorten all subheadings in the Results section to fewer than 60 characters including spaces.
*One subheading is at 60 characters and one at 61 – I cannot shorten further without altering meaning. The rest are <60 characters.*


LANGUAGE AND STYLE


* Please avoid using speech marks around words or phrases. In most cases they are unnecessary.

*removed*

* Please make sure that mathematical terms throughout your manuscript and Supplementary Information (including in figures, figure axes, and legends) conform strictly to the following guidelines. Equations should be supplied in editable format, and not as images. Scalar variables (e.g. x, V, χ) should be typeset in italic, whereas multi-letter variables should be formatted in roman. Constants (e.g. ħ, G, c) should be typeset in italics (the only exceptions being e, i, π, which should be typeset in Roman) and vectors (such as r, the wavevector k, or the magnetic field vector B) should be typeset in bold without italics. In contrast, subscripts and superscripts should only be italicised if they too are variables or constants. Those that are labels (such as the 'c' in the critical temperature, T_c, the 'F' in the Fermi energy, E_F, or the 'crit' in the critical current, I_crit) should be typeset in roman. To avoid doubt, unit dimensions should be expressed using negative integers
(e.g. kg m^-1 s^-2, not kg/ms^2) or the word 'per'.
*? single equation is ok as is*

* Please label equations sequentially as (1), (2), (3), etc. Subdivisions (1a, 1b, or 1.1, 1.2) are not permitted.

*done*

* Please use the format "Equation (1)", etc., to cite equations in the main text.

*done*


* Please ensure that +/- values are defined at the first point of use within the text and figure legends and numbers of replicates are given.
*done*

METHODS AND DATA

* Please rename the Methods section as "Methods".

*done*


* In the Methods section, please provide sufficient information such that the experiments could reasonably be reproduced without reference to other papers, and avoid use of the term "as described previously".

*done*


* Please include a complete list of all primers used in your study, including names and sequences, in either the Methods section or Supplementary Information.

*done*


* Please state the origins of all cell lines used in the Methods section (ATCC, vendor, catalogue number if relevant, etc.).

*done*


* All *Nature Communications* manuscripts must include a section titled "Data Availability" as a separate section after the Methods section and before the References. For more information on this policy, and a list of examples, please see <http://www.nature.com/authors/policies/data/data-availability-statements-data-citations.pdf>

*done*


* DATA SOURCES: We strongly encourage authors to deposit all new data associated with the paper in a persistent repository where they can be freely and enduringly accessed. We recommend submitting the data to discipline-specific, community-recognized repositories, where possible and a list of recommended repositories is provided here: <http://www.nature.com/sdata/policies/repositories>
If a community resource is unavailable, data can be submitted to generalist repositories such as figshare (<https://figshare.com/>) or Dryad Digital Repository (<http://datadryad.org/>). Please provide a unique identifier for the data (for example a DOI or a permanent URL) in the "Data Availability" section, if possible. If the repository does not provide identifiers, we encourage authors to supply the search terms that will return the data. For data that have been obtained from publically available sources, please provide a URL and the specific data product name in the "Data Availability" section. Data with a DOI should be included in the reference list and cited where relevant.
Please refer to our data policies here: <http://www.nature.com/authors/policies/availability.html>


END NOTES


* Please supply an "Author Contributions" section after the Acknowledgement section that refers to all authors.

*done*

* Please provide a "Competing Interests" section after the "Author Contributions" section that refers to all authors. If there are no competing interests, please add the statement "The authors declare no competing interests."

*done*


* Please ensure the references are in the standard Nature format and follow the sequence: author list, title of paper, name of journal, volume number, initial-final page numbers or article number (year). Please note that dois are required only for online-only publications and correct journal abbreviations should be given.

*done*


* References to websites are permitted only if they are in common use or curated. Please remove references to personal websites.
*removed*

DISPLAY ITEMS

* Please check whether your manuscript or Supplementary Information contain third-party images, such as figures from the literature, stock photos, clip art or commercial satellite and map data. We strongly discourage the use or adaptation of previously published images, but if this is unavoidable, please request the necessary rights documentation to re-use such material from the relevant copyright holders and return this to us when you submit your revised manuscript.

*n/a*


* Please include a brief title for all figure legends that summarises the whole figure and does not refer to specific panels.
*done*

* Please ensure that figure legend titles are brief - they should not occupy more than one line in the final proof.
*?okay*

* Please ensure that each display item is no larger than a single A4 portrait page (260x179 mm).
*done*

* Some figures in your paper include bar charts. Please overlay the corresponding data points (as dot plots) in the bar charts.
*done*

* Please ensure that all blots and gels are accompanied by the locations of molecular weight/size markers. Blots should be cropped such that at least one marker position is present. Please also supply uncropped and unprocessed scans of the most important blots in the Source Data file or as a supplementary figure in the Supplementary Information. This should be cited once in the Methods section.
*The uncropped gel has now been added as Supplementary Figure 4.*

* Please define any new abbreviations, symbols or colours present in your figures in the associated legends. Please do not use symbols in your legend, instead please write out the symbols in words (blue circles, red dashed line, etc.).
*done*

* In each figure and supplementary figure where error bars are used, they must be defined. One statement at the end of each figure is sufficient if the error bars are equivalent throughout the figure.
*done*

SUPPLEMENTARY INFORMATION

* We do not edit Supplementary Information files; they will be uploaded with the published article as they are submitted with the final version of your manuscript. Any tracked changes should be removed from the file and the file should be provided as a PDF file. Supplementary Figures do not need to be provided separately.


* The only section headings permitted in the Supplementary Information are Supplementary Figures, Supplementary Tables, Supplementary Methods, Supplementary Notes, Supplementary Discussion, Supplementary References. All other section headings and numbering should be removed or relabelled.
*done*

* In the Supplementary Information file and the main manuscript text, supplementary items must be labelled and cited using only the following formats: Supplementary Figure 1, Supplementary Table 1, Supplementary Methods, Supplementary Note 1, Supplementary Discussion, and Supplementary References. Please note the use of "Supplementary" and that we do not use the "S" prefix.
*done*

* Supplementary References should appear at the end of the Supplementary Information file, and should be self-contained and numbered from 1. References mentioned in both the main text and the Supplementary Information should be part of both reference lists so that the Supplementary Information does not refer to the reference list in the main paper and vice versa.
*done*

* Please supply legends for each Supplementary Movie/Audio/Data file in your cover letter (not in the Supplementary Information file). Please label each files as Supplementary Movie/Audio/Data 1, etc.
*done*

---------------------------------------------------------------------

* We encourage increased transparency in peer review by publishing the reviewer comments and author rebuttal letters of our research articles, if the authors agree. Such peer review material is made available as a supplementary peer review file. **Please state in the cover letter ‘I wish to participate in transparent peer review’ if you want to opt in, or ‘I do not wish to participate in transparent peer review’ if you don’t.** Failure to state your preference will results in delays in accepting your paper for publication.
Please note: we allow redactions to authors’ rebuttal and reviewer comments in the interest of confidentiality. If you are concerned about the release of confidential data, please let us know specifically what information you would like to have removed. Please note that we cannot incorporate redactions for any other reasons. Reviewer names will be published in the peer review files if the reviewer signed the comments to authors, or if reviewers explicitly agree to release their name. For more information, please refer to our FAQ page at:
<https://www.nature.com/documents/ncomms-transparent-peer-review.pdf>


* An updated editorial policy checklist that verifies compliance with all required editorial policies must be completed and uploaded with the revised manuscript. All points on the policy checklist must be addressed; if needed, please revise your manuscript in response to these points. Please note that this form is a dynamic "smart pdf" and must therefore be downloaded and completed in Adobe Reader, instead of opening it in a web browser.
Editorial policy checklist: <https://www.nature.com/authors/policies/Policy.pdf>
*done*

* An updated reporting summary must be completed and uploaded with the revised manuscript. All points on the reporting summary must be addressed; if needed, please revise your manuscript in response to these points. Please note that this form is a dynamic "smart pdf" and must therefore be downloaded and completed in Adobe Reader, instead of opening it in a web browser.
Reporting summary: <https://www.nature.com/authors/policies/ReportingSummary.pdf>
*done*

* Your paper will be accompanied by a two-sentence Editor's summary, of between 250-300 characters including spaces, when it is published on our homepage. Could you please approve the draft summary below or provide us with a suitably edited version.

EDITOR's SUMMARY:

Hypertrophic cardiomyopathy (HCM) leads to hyper-contractility of the heart and is often caused by mutations in human β-cardiac myosin. Here authors show that four separate β-cardiac myosin mutations can modulate myosin activity by disrupting intramolecular interactions.

*approved*


* As part of our efforts to communicate our content to a wider audience, we endeavour to highlight papers published in *Nature Communications* on the journal’s Twitter account ([@NatureComms](https://twitter.com/NatureComms)). If you would like us to mention authors, institutions or lab groups in these tweets, please provide the relevant twitter handles in your cover letter upon resubmission.
*done*

* In recognition of the time and expertise our reviewers provide to *Nature Communications*’s editorial process, as of November, 2018, we formally acknowledge their contribution to the external peer review of articles published in the journal. All peer-reviewed content will carry an anonymous statement of peer reviewer acknowledgement, and for those reviewers who give their consent, we will publish their names alongside the published article. For more information, please refer to our FAQ page at <https://www.nature.com/documents/ncomms-reviewer-information.pdf>
